# Assessing Metacognitive Regulation during Problem Solving: A Comparison of Three Measures

**DOI:** 10.3390/jintelligence11010016

**Published:** 2023-01-15

**Authors:** Cristina D. Zepeda, Timothy J. Nokes-Malach

**Affiliations:** 1 Department of Psychology and Human Development, Vanderbilt University, Nashville, TN 37235, USA; 2 Department of Psychology, Learning Research and Development Center, University of Pittsburgh, Pittsburgh, PA 15260, USA

**Keywords:** JOKs, learning, measurement, metacognition, metacognitive judgments, questionnaires, verbal protocols

## Abstract

Metacognition is hypothesized to play a central role in problem solving and self-regulated learning. Various measures have been developed to assess metacognitive regulation, including survey items in questionnaires, verbal protocols, and metacognitive judgments. However, few studies have examined whether these measures assess the same metacognitive skills or are related to the same learning outcomes. To explore these questions, we investigated the relations between three metacognitive regulation measures given at various points during a learning activity and subsequent test. Verbal protocols were collected during the learning activity, questionnaire responses were collected after the learning tasks but before the test, and judgments of knowing (JOKs) were collected during the test. We found that the number of evaluation statements as measured via verbal protocols was positively associated with students’ responses on the control/debugging and evaluation components of the questionnaire. There were also two other positive trends. However, the number of monitoring statements was negatively associated with students’ responses on the monitoring component of the questionnaire and their JOKs on the later test. Each measure was also related to some aspect of performance, but the particular metacognitive skill, the direction of the effect, and the type of learning outcome differed across the measures. These results highlight the heterogeneity of outcomes across the measures, with each having different affordances and constraints for use in research and educational practice.

## 1. Introduction

Metacognition is a multi-faceted phenomenon that involves both the awareness and regulation of one’s cognitions (Flavell 1979). Past research has shown that metacognitive regulation, or the skills learners use to manage their cognitions, is positively related to effective problem-solving (Berardi-Coletta et al. 1995), transfer (Lin and Lehman 1999), and self-regulated learning (Zepeda et al. 2015). Furthermore, these skills have been shown to benefit student learning across a variety of academic domains, including math, science, reading, and writing (Hacker et al. 2009). With research on metacognition advancing, multiple metacognitive skills have been proposed and evaluated, with researchers using different measures to assess each one (Azevedo 2020). Although many measures and approaches have been proposed (e.g., verbal protocols, questionnaires, metacognitive judgments), less work has compared and contrasted these different measures with one another. This has led to questions about the relations of the measures to one another and concerns about measurement validity (Veenman 2005; Veenman et al. 2003). To better understand metacognition conceptually and measure it practically, we need to compare how these different measures are similar to and different from one another.

In this work, we evaluate three types of metacognitive regulation measures: verbal protocols (e.g., students speaking their thoughts aloud during learning activities and then a researcher recording, transcribing, and coding those utterances for evidence of different metacognitive processes), a task-based questionnaire (e.g., asking students questions about how often they think they used different metacognitive processes during the learning task), and metacognitive judgments (specifically, judgments of knowing [JOKs]—a type of metacognitive judgment that asks students how confident they are about their answers on a test that is often based on content from a learning activity, sometimes referred to as retrospective confidence judgments). All three measures have been proposed to capture some aspect of metacognitive regulation. To evaluate the potential overlap of these measures, we conducted a theoretical analysis of each measure to better understand what it is intended to measure and how it has been typically used in research. We do so by reviewing the literature with consideration of each of the three measures in regard to their background theory, implications for what is learned, and attention to different aspects of validity. After this analysis, we investigate the measures in an empirical study, comparing and contrasting whether and how they are related to one another and learning outcomes during a problem-solving learning activity. Critically, this investigation has implications for practitioners trying to understand which aspects of their students’ metacognitive skills need support, as well as for theory and measurement development. Below, we first describe reasons why there might be some misalignment among the measures and then provide a detailed review of prior work using each type of measure and the validity of those measures.

### 1.1. Theory and Measurement: An Issue of Grain Size

One source of the variation in measurement is likely due to the variation in theories of metacognition (e.g., Brown 1987; Brown et al. 1983; Flavell 1979; Jacobs and Paris 1987; Nelson and Narens 1990; Schraw and Moshman 1995). Although most theories hypothesize that metacognition involves the ability to assess and regulate one’s thoughts, they differ in how they operationalize these constructs and their level of specificity (Pintrich et al. 2000; e.g., Nelson and Narens 1990; Schraw and Dennison 1994). Two common differences across models of metacognition are the number of constructs specified and the level of analysis at which those constructs are described. Relevant to this study, metacognitive regulation has been represented across models as containing a variety of skills, such as planning, monitoring, control, and evaluating.

**Figure 1:**
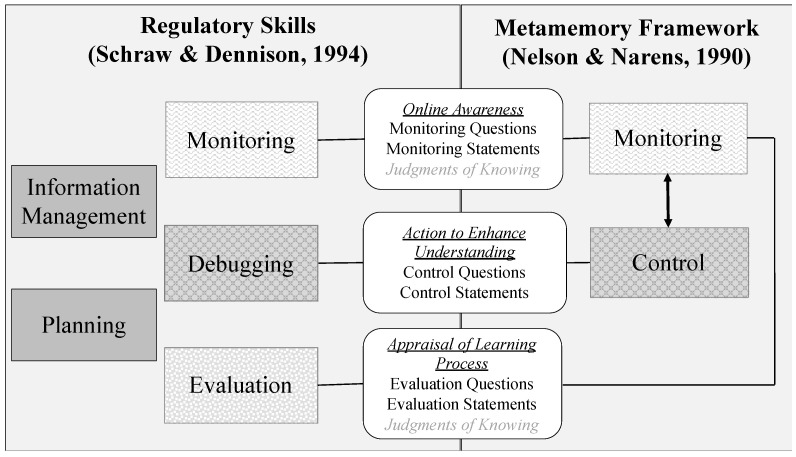
A comparison of the coarse-grain skills of two models that conceptualize metacognitive regulation. The gray and patterned rectangles represent the coarse-grain skills represented in each model. The rounded white rectangles connect to the coarse-grain skills that they are associated with for each of the models, highlighting the potential (mis)alignment between the constructs and measures. The rounded white rectangles also contain the definition for each of the coarse-grain skills and measures we aim to measure in this work. Note that judgments of knowing (JOKs) are shown in gray to represent the misalignment across the models with associations to evaluation for Schraw and Dennison (1994) and monitoring for Nelson and Narens (1990).

To illustrate the different number of constructs and the different levels of description, we compare a few models that conceptualize metacognitive regulation to one another. For example, Nelson and Narens’ (1990) model describes two higher-level constructs, whereas Schraw and Dennison’s (1994) model describes five constructs (see Figure 1 for an illustration). Nelson and Narens’ (1990) model consists of monitoring and control processes that assess the current state of working memory; it then uses that information to regulate and guide subsequent actions. These processes are described at a coarse grain level of analysis (see Figure 1), but the measurements of these constructs are operationalized at a more fine-grained level, focusing on different types of metacognitive judgments. Winne and Hadwin (1998) built upon this model and included additional higher-level metacognitive skills, such as planning and evaluating. Although Nelson and Narens’ (1990) model does contain aspects of planning (e.g., selection of processing) and evaluation (e.g., confidence in retrieved answers), these are included at the fine-grain level of description of monitoring and control and are not proposed as separate higher-level constructs.

Schraw and Dennison’s (1994) model also includes planning, monitoring, evaluating, as well as two additional higher-level skills, information management, and debugging. Similarly, Zimmerman’s (2001) self-regulated learning model includes the same metacognitive skills of planning, monitoring, and evaluation. Across these different models, each skill is hypothesized to have a distinct process that interacts with the other skills. To further illustrate some of these differences and similarities in the conceptualization of metacognitive regulation, in Figure 1, we compare Schraw and Dennison’s (1994) model with Nelson and Narens’ (1990) model. One clear difference between the two models is that JOKs are represented under monitoring in Nelson and Narens’ representation; however, given the definitions of monitoring and evaluation in Schraw and Dennison’s representation (as well as the other two models mentioned earlier), this might also be related to evaluation. This difference in particular highlights both the misalignment across the theories and the misalignment across theories and measurement.

This misalignment across theories and measurement is also seen with other measures. For example, although some researchers initially sought to capture specific metacognitive skills via a questionnaire, they often ended up combining them into a single factor due to the challenges of establishing each one as a separate construct (e.g., combining metacognitive skills such as monitoring and evaluating, among others, into a single component called metacognitive regulation—Schraw and Dennison 1994). Similarly, Pressley and Afflerbach (1995) had difficulty differentiating monitoring from control processes in verbal protocols and found that they tend to occur at the same time. The challenges in differentiating between the metacognitive skills of monitoring, control, and evaluating could also explain why other researchers have proposed fewer interactive skills (Howard-Rose and Winne 1993; Pintrich et al. 2000). In contrast, within hypermedia contexts, some researchers have been able to differentiate between specific, fine-grain skills, which they refer to as micro skills (e.g., learners questioning whether they understand the content) and larger-grain skills, which they refer to as macro skills (e.g., monitoring) (Azevedo and Witherspoon 2009; Greene and Azevedo 2009).

In this work, we examine the relation between theory and measurement with respect to a subset of metacognitive skills. This subset includes monitoring, control/debugging, and evaluating. We define monitoring as one’s awareness of one’s thinking and knowledge during the task, control/debugging as goal-directed activities that aim to improve one’s understanding during the task, and evaluation as an assessment of one’s understanding, accuracy, and/or strategy use once the task is completed. For example, if a student identifies what they do not understand (monitoring) while attempting to solve a problem, then they have an opportunity to fill the gap in their knowledge by seeking new information, rereading, summarizing the instructions, trying out new ideas, and so forth (control/debugging). Then, once the solution has been generated, they can reflect on their accuracy, as well as which strategies or knowledge they found most beneficial to prepare them for future tasks (evaluation). We chose this subset of metacognitive skills as they are commonly represented across theories of metacognition and measurements. Students are also more likely to engage in monitoring, control/debugging, and evaluation during problem-solving activities compared to other metacognitive skills such as planning, which appears to happen less frequently, as students often just dive right into solving the problem (e.g., Schoenfeld 1992).

### 1.2. Relation among Measures

**Figure 2:**
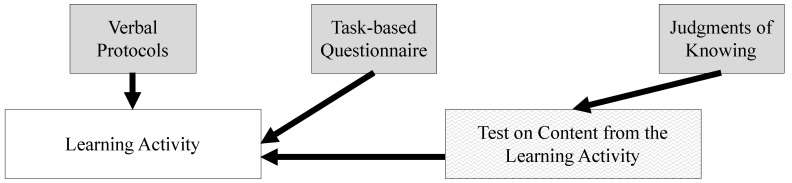
Visual representation of our across-methods-and-time design. The arrows indicate what each measure references. The verbal protocols were collected as a concurrent measure in reference to the learning activity. The task-based questionnaire was collected as a delayed retrospective measure in reference to the learning activity. The JOKs were collected as an immediate retrospective measure in reference to the test that was based on the learning content. Note, however, that the JOKs may act more like concurrent measures, as they are generated with the information still present (e.g., problem content); therefore, the box with JOKs overlaps more with the test on the learning activity, whereas the task-based questionnaire does not overlap with the learning activity.

In addition to the issues of grain size, there are two additional factors that differ across the measures. These factors concern *when* (e.g., prospective, concurrent, or retrospective) and *how* (e.g., think aloud vs. questionnaire vs. judgment) metacognition is assessed. Concurrent or “online” measures such as verbal protocols (e.g., Chi et al. 1989) attempt to examine people’s metacognition *as it is occurring,* whereas retrospective measures such as questionnaires (e.g., Schraw and Dennison 1994) and JOKs (i.e., retrospective confidence judgments; see Dunlosky and Metcalfe 2009 for an overview) evaluate metacognition *after* the skills have been employed and/or a solution has been generated or the answer has been given. Unlike a task-based questionnaire, which typically takes place at a longer interval after completing a learning activity, JOKs that assess one’s confidence on test items take place immediately after each problem is solved. Therefore, in Figure 2, there is more overlap between the JOKs and the test than there is between the task-based questionnaire and the learning activity. A key difference between the timing of all these measures is that, in contrast with the retrospective measures, concurrent verbal protocols allow access to the contents of working memory without having to rely on one’s long-term memory (Ericsson and Simon 1980). Given that JOKs occur after a problem is solved, but also while the information is still present, they may act more like a concurrent measure than a retrospective measure. See Figure 2 for a visual representation of where some of these measures take place during the learning and assessment sequence that we used in the present study.

Critically, few studies have directly compared these measures to one another. Those that have, have shown that student responses to questionnaires rarely correspond to concurrent measures (Cromley and Azevedo 2006; Van Hout-Wolters 2009; Veenman 2005; Veenman et al. 2003; Winne and Jamieson-Noel 2002; Winne et al. 2002). For example, Veenman et al. (2003) found weak associations (*r*’s = −.18 to .29) between verbal protocols and a questionnaire assessing students’ metacognitive study habits. Van Hout-Wolters’ (2009) work revealed similar findings, in which correlations between verbal protocols and dispositional questionnaires were weak (*r*’s = −.07 to .22). In addition, Zepeda et al. (2015) found that students who received metacognitive training differed from a comparison condition in their accuracy in discriminating the metacognitive accuracy in their JOKs, but *not in* their general questionnaire responses. Schraw and Dennison (1994) and Sperling et al. (2004) showed similar findings, in which student accuracy regarding their JOKs was not related to their responses on the Metacognitive Awareness Inventory’s (MAI) metacognitive regulation dimension. The lack of associations among the different metacognitive measures may be due to the measures assessing different processes, an imprecise measure, or a combination of the two. Veenman et al. (2006) suggested that researchers should use a multi-method design to explicitly compare different methodologies and determine their convergent and external validity.

### 1.3. Relations to Robust Learning

Another way to examine the similarity of the measures is to examine whether they predict similar learning outcomes (e.g., external validity). To what degree do these different measures of the same construct predict similar learning outcomes? Prior research provides some evidence that metacognition is related to school achievement (e.g., grades or GPA) and performance on tests (e.g., quizzes, standardized assessments). However, no work has examined whether all three measures of the same construct predict the same type of learning outcome. Therefore, we investigated whether the different measures predicted different types of robust learning outcomes.

**Figure 3:**
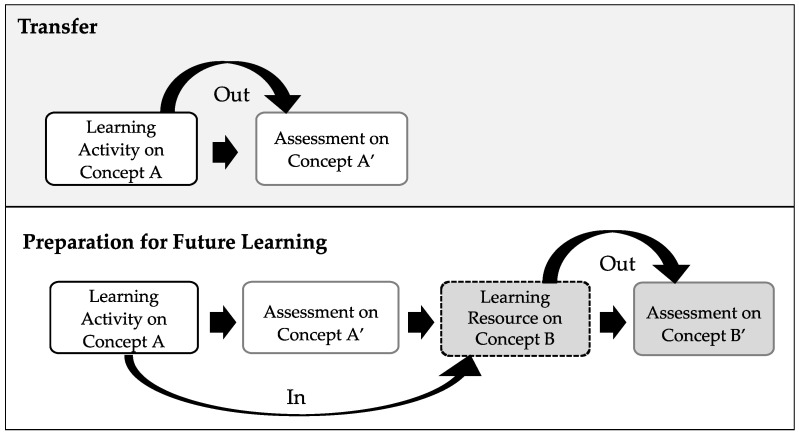
A comparison of the flow of information and knowledge between transfer and PFL, as derived from Bransford and Schwartz (1999) and Schwartz et al. (2005). The top light-gray box represents transfer, and the bottom white box represents PFL. “Out” means that the knowledge learned is then demonstrated on an outside assessment. “In” means the learner takes in the information from the learning activity to inform how they interpret later information. The A’ and B’ on the assessment designate that the problems are not identical to the original problems presented in the learning activity.

Robust learning is the acquisition of new knowledge or skills, which can be applied to new contexts (transfer) or prepare students for future learning (PFL) (Bransford and Schwartz 1999; Koedinger et al. 2012; Schwartz et al. 2005; Richey and Nokes-Malach 2015). Transfer is defined as the ability to use and apply prior knowledge to solve new problems and PFL is defined as the ability use prior knowledge to *learn* new material (see Figure 3 for a comparison). For example, to assess transfer in the current study, learners attempt to apply knowledge (e.g., concept A) acquired from a statistics learning activity to new questions on a post-test that address the same concept (e.g., concept A’). Schwartz et al. refer to this process as ’transferring out’ knowledge from learning to test. To assess PFL, an embedded resource (Concept B) is incorporated into the post-test, in which learners have to apply what they learned in the earlier learning activity (i.e., their prior knowledge, Concept A) to understand the content in the resource. This is what Schwartz et al. refer to as ‘transferring in’. Then, that knowledge is assessed with a question to determine how well the students learned that information (i.e., testing with Concept B’). To our knowledge, there is no work examining the relation between metacognition and PFL using these different metacognitive regulation measures. To gain an understanding of how these measures have been related to different learning outcomes, we surveyed the literature.

#### 1.3.1. Verbal Protocols and Learning

Past work has examined the relation of verbal protocols to different types of learning. For example, Van der Stel and Veenman (2010) found that increased use of metacognitive skills (e.g., planning, monitoring, and evaluating) was associated with better near transfer (e.g., performance on isomorphic problems with the same problem structure but different surface features). In other work, Renkl (1997) found that the frequency of positive monitoring statements (e.g., “that makes sense”) was unrelated to transfer performance, but the frequency of negative monitoring statements (e.g., “I do not understand this”) was negatively related to transfer. This result shows that different types of metacognitive phenomena are differentially related to transfer. In this case, monitoring behaviors can be useful for identifying when a learner does not understand something.

#### 1.3.2. Questionnaires and Learning

Metacognitive questionnaires are typically used to capture the relation between metacognitive skills with measures of student achievement as assessed by class grades, GPA, or standardized tests (Pintrich and De Groot 1990; Pintrich et al. 1993; Sperling et al. 2004). However, a focus on achievement measures makes it difficult to determine how much and what type of knowledge a student gained because the measures are coarse grained and often do not account for prior knowledge. For example, class grades (which determine GPA) typically include other factors in addition to individual learning assessments, such as participation and group work. Unlike prior work with verbal protocols, research using questionnaires has not evaluated the relations of metacognitive skills and different types of learning outcomes, such as transfer or PFL.

#### 1.3.3. Metacognitive Judgments—JOKs and Learning

Judgments of knowing (JOKs) have typically been used in paired-associate learning paradigms (Winne 2011). However, some work has examined JOKs and their relation to test performance and GPA (Nietfeld et al. 2005, 2006). For example, Nietfeld et al. (2005) found that students’ JOKs were positively related to learning outcomes across different tests (that included transfer items), even when controlling for GPA.

#### 1.3.4. Summary of the Relations to Robust Learning

From this brief survey of the prior literature, we see that different metacognitive measures have been related to different types of learning outcomes. Questionnaires have primarily been related to achievement outcomes (e.g., grades and GPA), whereas verbal protocols and JOKs have been related to multiple learning outcomes, including achievement and transfer. This variation makes it difficult to determine whether these measures predict the same types of learning. To gain a better understanding of how metacognition is related to learning, we examine the relations among all three measures to transfer and PFL. These empirical and theoretical challenges have direct implications for determining measurement validity.

### 1.4. Measurement Validity

Given the different approaches used across the three metacognitive measures and drawing inspiration from Pintrich et al.’s (2000) review, we used aspects of Messick’s (1989) validity framework to structure our review for the validity and scope of each measure. The components of measurement validity that we focus on include substantial validity, external validity, content validity, generality of the meaning (generality for short), and relevance and utility (utility for short). Substantial validity concerns whether the measure produces the predicted structure of the theoretical constructs (e.g., the type and number of metacognitive skills). External validity concerns the predictive or convergent relations to variables that the theory predicts (e.g., the relation to similar types of learning outcomes and the relation between metacognitive measures). Content validity concerns whether the measure is tailored to a specific activity or material. Generality concerns the applicability of the measure to different populations, while utility examines the ease of implementation. Below, we describe each metacognitive measure and their alignment with each of the five aspects of validity.

#### 1.4.1. Validity of Verbal Protocols

Verbal protocols provide fine-grained verbal data to test hypotheses about what and how metacognition is used when a participant is engaged in some learning or problem-solving activity. However, the level of theoretical specificity depends on the research goals of the work, the research questions asked, and the coding rubrics constructed. For example, Renkl (1997) only examined negative versus positive monitoring, whereas other verbal protocol analyses have attempted to create a detailed taxonomy for evaluating the metacognitive activity of a learner, regardless of the valence (Greene and Azevedo 2009; Meijer et al. 2006). Although Meijer et al. (2006) originally sought to develop a fine-grain taxonomy, due to difficulties obtaining interrater reliability, they condensed their codes into fewer, more generalized aspects of metacognition. These examples reveal that verbal protocols have not arrived at a consensus for the level of analysis with existing protocols, revealing mixed results for the substantive validity of this approach.

Verbal protocols also have mixed results regarding external validity, as they have been shown to correlate with learning outcomes in some studies (e.g., Van der Stel and Veenman 2010), but not others (Meijer et al. 2012; Renkl 1997). However, this might be attributed to the way in which the verbal protocols were coded. Some coding rubrics differ in whether they code for the quality of metacognition (e.g., accuracy in application) versus the quantity of a specific metacognitive activity (e.g., the frequency of occurrence) (Meijer et al. 2012).

Within a specific coding rubric, there is evidence that verbal protocols have some content validity, as it is domain general. Veenman et al. (1997) found that the same coding rubric could be applied across three domains and was predictive of learning outcomes within each domain. Verbal protocols have also been successfully employed with a variety of populations (e.g., Veenman et al. 2004) and can be applied to a variety of contexts and tasks. They have been used in physics (Chi et al. 1989), biology (Gadgil et al. 2012), probability (Renkl 1997), and reading (Pressley and Afflerbach 1995), among others. Thus, we reveal the flexibility in applying this approach across different content and contexts.

One drawback of verbal protocols is that they take a substantial amount of time to administer and evaluate. Instead of administering the measurement to groups of students, researchers typically focus on one student at a time because of the challenges of recording multiple speakers and potential verbal interference across speakers in the same room. These protocols also require more time to transcribe and code, making this a time-consuming task for researchers and practically challenging to use in the classroom. Although think-aloud protocols are more difficult to employ in classrooms, they provide benefits to researchers, such as a fine-grained source of trace data (Ericsson and Simon 1980). So, while there is utility in the fine-grain products, there is a lack of practical utility in classrooms.

#### 1.4.2. Validity of Questionnaires

Questionnaires are often used to determine the degree to which students perceive using various metacognitive skills. The majority of questionnaires ask students to report on their dispositional use of the skills, although a few are specific to a task or context. The similarity between the structure of the measurement and theory is not well aligned. Although many questionnaires attempt to assess fine-grain distinctions between metacognitive skills, they often have difficulty doing so empirically. For example, Schraw and Dennison (1994) originally sought to capture five distinct metacognitive skills within the MAI; however, the results revealed only a single factor. Similar to verbal protocols, this misalignment reveals that questionnaires have not arrived at a consensus for the level of analysis with existing questionnaires, revealing mixed results for the substantive validity of this approach.

In contrast, there is more evidence for the external validity of questionnaires. Prior work has shown that questionnaires relate to other variables predicted by metacognitive theory, such as achievement (Pintrich and De Groot 1990; Pintrich et al. 1993) as well as convergence with similar questionnaires assessing similar processes (Sperling et al. 2004; Muis et al. 2007). For example, Sperling et al. (2004) found that the Regulation of Cognition dimension of the MAI was related to the Metacognitive Self-Regulation scale of the Motivated Strategies for Learning Questionnaire (MSLQ; Pintrich et al. 1991) (r = .46).

The content validity of a questionnaire depends on its intended scope. Some questionnaires are designed to capture the general use of metacognitive skills such as the MAI or MSLQ. Other questionnaires assess metacognitive skills for a particular task. For example, work by Van Hout-Wolters (2009) demonstrated that task-based measures have a stronger positive relation to verbal protocols than dispositional questionnaires. It is difficult to assess the strength of these different types of questionnaires because dispositional questionnaires typically focus on a generalization of the skills over a longer time-period than task-based questionnaires.

Additionally, metacognitive questionnaires have been reliably adapted to serve a variety of ages (e.g., Jr. MAI; Sperling et al. 2002). Of particular interest to educators and researchers is the utility of the measure with the ease of administering and scoring the instrument. Researchers have sought to develop easy-to-use retrospective questionnaires that take just a few minutes to complete. Perhaps the ease of this measure is the reason why there are many questionnaires aimed at capturing different types of content, making it difficult to assess the validity of such measures.

#### 1.4.3. Validity of Metacognitive Judgments—JOKs

JOKs assess students’ accuracy in their monitoring of how well they know what they know after they have solved a problem or answered a question. Although often referred to as a monitoring component, some work also refers to these judgments as an evaluative skill (e.g., Winne 2011). Therefore, JOKs might measure one or both monitoring and evaluating skills. In some studies, these skills are collapsed together (e.g., Kistner et al. 2010). JOKs are one of many types of metacognitive judgments (see Alexander 2013 for an overview). We used JOKs because there has been some evidence suggesting that they have stronger relations to performance outcomes in comparison to the other types of metacognitive judgments (Hunter-Blanks et al. 1988). JOKs also allowed us to gather multiple observations during an assessment, whereas we would have been limited in the number of observations for the other types of judgments given the nature of the learning task (see below for a description of the learning task).

Different types of calculations have been applied to determine the accuracy and consistency of student judgments (see Schraw 2009; Schraw et al. 2013). The prior literature has shown some evidence for substantive validity in that it is designed to capture one to two metacognitive skills, referred to as monitoring and evaluating. However, this structure may differ depending on the calculations used to assess different types of accuracy (see Schraw 2009 for a review). JOKs also have some evidence of external validity, as Nietfeld et al. (2005, 2006) showed that student judgments were related to learning performance and GPA.

The content validity of JOKs is unclear. Some work has demonstrated it is domain general (Mazancieux et al. 2020; Schraw 1996; Schraw et al. 1995) and other work has shown it is domain specific (Kelemen et al. 2000). For example, Schraw (1996) showed that when controlling for test difficulty, confidence ratings from three unrelated tests (math, reading comprehension, and syllogism) were moderately related to each other (average *r* = .42). More recently, work has compared the types of calculations that have been applied to JOKs (Dentakos et al. 2019). For example, calibrations of JOKs are positively related across tasks, but the resolution of the JOKs (e.g., relative accuracy and discrimination) are not positively related across tasks, suggesting that the type of calculation applied to determine one’s accuracy has implications for when and how JOKs are related. Regardless of these limitations, JOKs have also been applied to multiple domains (e.g., physics, general facts) and age groups (Dunlosky and Metcalfe 2009).

In terms of utility, JOKs are moderately easy to implement. It takes more time to determine the accuracy calculations of these judgments than it does to evaluate questionnaire responses, but it is not as time intensive as verbal protocols. Thus, from a practical standpoint, there is utility in the administration of JOKs, but the utility in applying the calculations is challenging, as it requires additional time to apply those calculations, as well as the knowledge of how and what types of calculations to apply.

Drawing from Zepeda et al. (2015), we focus on the relation between three types of JOK calculations: absolute accuracy, gamma, and discrimination. They found differences in an experimental manipulation for one type of calculation (discrimination) but not others (absolute accuracy and gamma), suggesting that they captured different metacognitive processes. Therefore, in this study, we employ three different types of calculations: absolute accuracy and two measures of relative accuracy, gamma, and discrimination. Absolute accuracy compares judgments to performance, whereas Gamma evaluates confidence judgment accuracy on one item relative to another (Nelson 1996). Schraw (1995) suggested that since there is not a one-to-one relation between gamma and absolute accuracy, research should report both. Discrimination examines the degree to which students can distinguish their confidence regarding an incorrect or correct performance (Schraw 2009). Positive discrimination indicates that a learner gave higher confidence ratings for correct trials compared to incorrect trials, a negative value indicates higher confidence ratings for incorrect trials compared to correct trials, and a zero indicates no relation between the two. It can be interpreted that those with positive discrimination are aware of their correct performance. In addition to these calculations, we also examined average JOK ratings, given that students are typically poor at calibrating their understanding when the task is difficult (Howie and Roebers 2007).

#### 1.4.4. Summary of Measurement Validity

The validity across the three types of measurement reveals two consistent patterns, such that they all have been applied to different age groups (generality), and they tend to have mixed or only some support for their substantive validity. For the remaining three types of validity, different patterns emerge. Both questionnaires and JOKs have evidence of external validity and their content validity tends to be more sensitive to context. In contrast, for verbal protocols, there is mixed support for external validity and evidence of content validity. Additionally, all three measurements range in their ease of implementation (their utility) such that questionnaires are more easily applied and scored in educational contexts than JOKs, and both are easier to implement than verbal protocols. Given this landscape, we paid particular attention to the design and development of each measure, especially their alignment with theory (i.e., substantive validity) and their framing to content (e.g., using a task-based questionnaire versus a general one and examining different calculations of JOK accuracy).

### 1.5. Underlying Processes of the Measures

In addition to their relations to learning outcomes and past work evaluating their validity and scope, these measures likely capture similar and different processes. For example, for the monitoring skills, all three measures likely capture some aspect of monitoring, such as reflecting on one’s use of monitoring during a task-based questionnaire, the actual verbalization of monitoring, and the monitoring that contributes to one’s JOKs. At the same time, each of these measures might also reflect other processes. Reporting one’s use of monitoring requires the person to be aware of their use of monitoring, to monitor their monitoring, and rely on their long-term memory, whereas the verbal protocols capture the monitoring as it unfolds. These verbal protocols also likely contain more information about how the monitoring unfolds and might be more accurate at distinguishing between monitoring, control/debugging, and evaluating one’s learning process. In contrast, self-reporting on these skills might have more cross-over effects when students reflect on using these skills and determining the boundaries between them. The JOKs are similar to the task-based questionnaire, such that they may rely on monitoring the monitoring that took place during the learning task and one’s long-term memory of that experience, but they are different in that JOKs mainly involve monitoring one’s monitoring during the test. Some recent work supports the idea that there may be different monitoring skills at play among the measures. For example, McDonough et al. (2021) revealed that there appear to be two types of monitoring skills among metacognitive judgments: monitoring skills that occur during the encoding stage versus monitoring skills that occur at the retrieval stage, such that they rely on different pieces of information and cues (e.g., the difficulty of the learning task versus post-test).

As described when comparing the monitoring task-based questionnaire and monitoring statements, the control/debugging skills represented in the task-based questionnaire and the verbal protocols likely have similar overlaps, with some additional differences. Reporting one’s use of control/debugging requires them to be aware and monitor their control/debugging while also relying on their long-term memory. In contrast, the verbalizations capture the control/debugging as it unfolds. The degree of their need to control/debug their learning might also have implications for their reports on the questionnaire, such that in their reports, they might focus on the quantity as well as the productivity of their controlling/debugging.

Evaluating can also be captured across all three types of measures, but more directly by the verbal protocols and the task-based survey. For instance, the processes captured in the task-based questionnaire require learners to be aware of their evaluation process and know of the boundary between the skills. The verbal protocols more directly capture the evaluations as they occur and allow for a potentially more accurate differentiation between monitoring and evaluating. Additionally, the JOKs require students to reflect on their current understanding (i.e., monitoring) but also include aspects in which they evaluate how well they solved the present problem and learned the material during the learning activity. Thus, those measures may be related as well.

Given the different processes, boundaries, and demands of these different types of measures that aim to capture the same set of metacognitive skills, some aspects suggest that they should be related across the measures. Other aspects suggest that these measures may not be well aligned with one another because of the different processes that are required for each skill and measurement type. Therefore, the question remains: when the measures are developed to capture the same metacognitive skills, do they have similar relations to each other and learning outcomes?

### 1.6. Current Work

In this work, we assessed the relations among three metacognitive regulation measures: a retrospective task-based questionnaire, concurrent verbal protocols recorded during a learning activity, and JOKs elicited during a posttest (outlined in Table 1). The overall goal of this study was to investigate how these measures related to each other and to determine the degree to which they predict the similar outcomes for the same task.

Therefore, we hypothesized that:

**H1a** **and H1b.** *Given that prior work tends to use these measures interchangeably and that they were developed to capture the same set of metacognitive skills, one hypothesis is that they will be positively related, as they assess similar metacognitive processes. Monitoring and evaluating assessed by JOKs will have a small positive association with the monitoring and evaluating assessed by the verbal protocols and the task-based questionnaire (rs between .20 and .30), but the associations for one type of skill might be higher than the other. This relation is expected to be small given that they occur at different time points with different types of activities, although all on the same learning content. We also predict a moderate relation between the verbal protocols and the task-based questionnaire for monitoring, control/debugging, and evaluating (rs between .30 and .50), which would be consistent with past work examining the relations between questionnaire and verbal protocols by Schellings and Van Hout-Wolters (2011) and Schellings et al. (2013). This relation is larger given that both measures are used in the same learning task but at slightly different time points. Alternatively, given the lengthy review we conducted showing that these measures are often not positively related with each other and that the measures themselves may require different processes, these measures might not be related to one another, even after being carefully matched across the metacognitive skills.*

**H2.**  *Although there are nuances between each of the measures, the prior work we reviewed suggests that they all should predict performance on learning, transfer, and PFL.*

Prior studies examining metacognition tend to utilize tell-and-practice activities in which students receive direct instruction on the topic (e.g., Meijer et al. 2006). In contrast, we chose a structured-inquiry activity, as it might provide more opportunities for students to engage in metacognitive regulation (Schwartz and Bransford 1998; Schwartz and Martin 2004). A core feature of these activities is that students try to invent new ways to think about, explain, and predict various patterns observed in the data. In the task we chose, students attempt to solve a challenging statistics problem in which they have an opportunity to monitor their progress and understanding, try out different strategies, and evaluate their performance. Although there is controversy in the learning sciences about the benefits of inquiry-based instruction (Alfieri et al. 2011), several research groups have accumulated evidence of the benefits of these types of structured inquiry activities in math and science domains (e.g., Belenky and Nokes-Malach 2012; Kapur 2008; Roll et al. 2009; Schwartz and Martin 2004). For example, these activities have been shown to engage students in more constructive cognitive processes (Roll et al. 2009) and to facilitate learning and transfer (Kapur and Bielaczyc 2012; Kapur 2008, 2012; Roll et al. 2009).

## 2. Materials and Methods

### 2.1. Participants

Sixty-four undergraduates (13 female, 51 male) enrolled in an Introductory Psychology course at a large Mid-Atlantic university participated in the study. All students consented to participate in the study and received credit for their participation. We excluded data from 19 students from the analyses, as they were able to correctly solve for mean deviation and/or standard deviation on the pre-test, which were the two mathematical concepts to be learned during the learning activity. The remaining 45 students (9 female, 36 male) were included in the analyses, as they still had an opportunity to learn the material. Within this sample, student GPAs included a broad range, with students self-reporting below a 2.0 (4.4%), 2.0–2.5 (20%), 2.5–3.0 (28.9%), 3.0–3.5 (24.4%), and 3.5–4.0 (22.2%). Within this sample, 77.8% of the students identified as white, 6.7% as African American, 6.7% as Biracial, 4.4% as Hispanic, 2.2% as Asian Indian, and 2.2% did not specify.

### 2.2. Design

**Figure 4:**
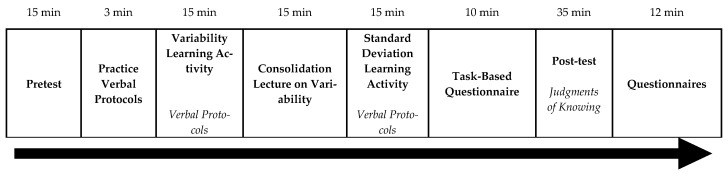
Design summary. The three metacognitive measures captured in this work are italicized. The arrow shows the direction of the ordered activities.

Using an across-method-and-time design, we recorded student behaviors with video-recording software during a learning activity and collected student responses to a task-based questionnaire and JOKs. See Figure 4 for an overview of the experimental design, materials, and procedure.

### 2.3. Materials

The materials consisted of a pre-test, a learning task, a task-based questionnaire, a post-test, and an additional questionnaire that captured demographic information. The learning task was divided into three segments: an invention task on variability, a lecture on mean deviation, and a learning activity on standard deviation. These were identical to those used by Belenky and Nokes-Malach (2012), which were adapted from Schwartz and Martin (2004). Parts of the questionnaires assessed student metacognition, motivation, and cognitive processes; however, for this paper, we focus only on the metacognitive components.

#### 2.3.1. Learning Pre-Test

The pre-test was used as a screening tool to remove data from participants who already knew how to solve mean, mean deviation, and standard deviation problems. These items were adapted from Belenky and Nokes-Malach (2012) and Schwartz and Martin (2004). All students completed a pre-test with three types of items targeting procedural and conceptual knowledge. All items were scored as either correct (1) or incorrect (0). Two questions assessed basic procedural knowledge of mean and mean deviation, and one assessed a conceptual problem that is matched to a preparation for future learning problem in the post-test (PFL; Bransford and Schwartz 1999).

#### 2.3.2. Learning Task

The learning task consisted of two activities and a lecture. The first learning activity was based on calculating variability. Students were asked to invent a mathematical procedure to determine which of four pitching machines was most reliable (see Belenky and Nokes-Malach 2012, Figure 4, p. 12; Schwartz and Martin 2004, p. 135). The consolidation lecture provided an example that explained how to calculate variability using mean deviation and two practice problems with feedback on how to correctly solve the problems. The second activity asked students to invent a procedure to determine which of two track stars on two different events performed better (Bill on the high jump versus Joe on the long jump). Students received scratch paper and a calculator.

*Scoring of the learning activities*. Learning materials were evaluated based on the use of correct procedures and the selection of the correct response. Since students could determine the correct answer based on evaluating the means, we coded for every step students took and their interpretations of their final answers. For the variability activity, students could receive a total of 4 points. They received 1 point for calculating the mean, 1 for subtracting the numbers from the mean and taking the absolute value, 1 for taking the mean of those numbers, and 1 for stating that the Fireball Pitching Machine was the most reliable. For the second activity, the standardization activity, students could receive a total of 5 points. They received 1 point for calculating the mean, 1 for subtracting the numbers from the mean and squaring that value, 1 for taking the mean of those numbers, 1 for taking the square root of that value, and 1 for stating that Joe was more reliable.

#### 2.3.3. Learning Post-Test

Similar to the pretest, many of the post-test items were identical to or adapted from Belenky and Nokes-Malach (2012), Gadgil (2014), and Schwartz and Martin (2004). The post-test contained seven items that measured students’ conceptual and procedural knowledge of the mean deviation. It also assessed students’ abilities to visually represent and reason about data. These items assess a variety of different types of transfer such as near and immediate (e.g., Nokes-Malach et al. 2013). For this work, we do not analyze these levels of transfer separately as there are not enough items for each transfer type to effectively examine outcomes.

Within the assessment, there was also a PFL problem that evaluated students’ abilities to apply information from an embedded resource to this standard deviation problem. The embedded learning resource was presented as a worked example in the post-test and showed students how to calculate a standardized score with a simple data set which was identical to Belenky and Nokes-Malach (2012, Figure 8, p. 16; adapted from Schwartz and Martin 2004, pp. 177–78). This resource also gave another simple problem using standardized scores. The PFL transfer problem appeared five problems after the worked example. The problem was presented later in the post-test so that the application of the information was not due to mere temporal proximity (i.e., the next problem), but instead, it required that students to notice, recall, and apply the relevant information at a later time. The PFL problem required students to determine which value from two different distributions was more impressive than the other. During the post-test, students were also asked to respond to a JOK for each problem in which they rated how confident they were in their answer from 1 being *not at all confident* to 5 being *very confident*.

Scoring of post-test items. Each item was coded for accuracy. The post-test comprised two types of problems: 6 transfer items focused on solving the correct procedure and understanding the concepts of mean deviation (α = .39) and 1 PFL problem. Two transfer problems involved the use of the correct procedure in which a correct response was coded as 1, and an incorrect response was coded as a 0. The other four transfer problems involved reasoning and were coded for the amount of detail within their reasoning. Each of these conceptual problems included different types of reasoning. One point was granted for a complete understanding of the concept or either a .67, .50, .33 for partial understanding (dependent on how many ideas were needed to represent a complete concept) or a 0. The post-test transfer items were scored out of a total of 6 points. The PFL problem was scored as correct (1) or incorrect (0).

#### 2.3.4. Verbal Protocols

To provide practice with talking aloud, we included a 3 min activity where participants solved multiplication problems. Specifically, participants were told, “As you go through the rest of the experiment, there are going to be parts where I ask you to talk aloud, say whatever you are thinking. It is not doing any extra thinking, different thinking, or filtering what you say. Just say whatever it is you are naturally thinking. We’re going to record what you say in order to understand your thinking. So, to practice that, I will give you some multiplication problems; try solving them out loud to practice.” Then, the experimenter was instructed to give them feedback about how they were talking aloud with prompts such as,” That is exactly right, just say what you’re thinking. Talk aloud your thoughts.” Or “Remember to say your thoughts out loud” or “Naturally say whatever you are thinking, related or unrelated to this. Please do not filter what you’re saying.” Once participants completed the practice talking aloud activity, they were instructed to talk aloud for the different learning activities.

*Processing and coding of the verbal protocols*. To capture the metacognitive processes, we used prior rubrics for monitoring, control/debugging, and evaluating (Chi et al. 1989; Gadgil et al. 2012; Renkl 1997; see Table 2). We also coded for two distinct types of debugging—conceptual error correction and calculation error correction. These were coded separately, as these types of corrections might be more directly related to better performance. Students who focus on their conceptual or procedural (calculation) understanding are aiming to increase a different type of understanding than those who are rereading or trying out other strategies. Those who reread and try out different strategies are still on the path of figuring out what the question is asking them to achieve, whereas those who are focusing on conceptual and calculation errors are further in their problem-solving process. Critically, we coded for the frequency of each metacognitive process as it aligned with prior rubrics that have measured verbal protocols in the past. We hypothesized that the first learning activity would have representative instances of metacognitive regulation, since it was an invention task.

All videos were transcribed and coded from the first learning activity on variability. Statement length was identified by clauses and natural pauses in speech. Then, two coders independently coded 20% of the data and reached an agreement as examined by an inter-coder reliability analysis (*k* > .7). The coders discussed and resolved their discrepancies. Then, they independently coded the rest of the transcripts. The verbal protocol coding was based on prior rubrics and is represented with examples from the transcripts in Table 2. Due to an experimental error, one participant was not recorded and was therefore excluded from all analyses involving the verbal protocols. For each student, we counted the number of statements generated for each coding category and divided this number by their total number of statements. On average students generated 58.79 statements with much variation (*SD* = 34.10). Students engaged in monitoring the most (*M* = 3.05 statements per student) followed by evaluation (*M* = 2.71 statements per student). Students rarely employed control/debugging, conceptual error correction, and calculation error correction (*M* = .23, .05, and .61, respectively). Therefore, we combined these scores into one control/debugging verbal protocol code (*M* = .88 statements per student).

We also examined the relations between the total number of statements generated (i.e., verbosity) and the number of statements for each type of metacognitive category. The amount students monitored (*r* = .59, *p* < .001), control/debugged (*r* = .69, *p* < .001), and evaluated (*r* = .72, *p* < .001) their understanding was related to the total number of utterances. Given this relationship, we divided each type of verbal protocol by the total number of utterances to control for the number of utterances.

#### 2.3.5. Task-Based Metacognitive Questionnaire

We adapted questionnaire items from previously validated questionnaires and verbal protocol coding rubrics (Chi et al. 1989; Gadgil et al. 2012; Renkl 1997) as indicated in Table 3. Informed by this research and Schellings and Van Hout-Wolters’ (2011) in-depth analysis of the use of questionnaires and their emphasis on selecting an appropriate questionnaire given the nature of the to-be-assessed activity, we created a task-based questionnaire and adapted items from the MAI, MSLQ, Awareness of Independent Learning Inventory (AILI, Meijer et al. 2013), a problem-solving based questionnaire (Howard et al. 2000; Inventory of Metacognitive Self-Regulation [IMSR] that was developed from the MAI and Jr. MAI as well as Fortunato et al. 1991), and a state-based questionnaire (O’Neil and Abedi 1996; State Metacognitive Inventory [SMI]). In total, there were 24 metacognitive questions: 8 for monitoring, 9 for control/debugging, and 7 for evaluation. Students responded to each item using a Likert scale ranging from 1, *strongly disagree,* to 7, *strongly agree*. All items and their descriptive statistics are presented in Table 3. We chose to develop and validate a task-based metacognitive questionnaire for three reasons. First, there is mixed evidence about the generality of metacognitive skills (Van der Stel and Veenman 2014). Second, there are no task-based metacognitive measures for a problem-solving activity. Third, to our knowledge, no existing domain-general questionnaires reliably distinguish between the metacognitive skills of monitoring, control/debugging, and evaluation.

To evaluate the substantive validity of the questionnaire, we used a second-order CFA model consisting of three correlated factors (i.e., monitoring, control/debugging, and evaluation) and one superordinate factor (i.e., metacognitive regulation) with MPlus version 6.11. A robust weighted least squares estimation (WLSMV) was applied. Prior to running the model, normality assumptions were tested and met. The resulting second-order CFA model had an adequate goodness of fit, CFI = .96 TLI = .96, RMSEA = .096, *X*^2^ (276) = 2862.30, *p* < .001 (Hu and Bentler 1999). This finalized model also had a high internal reliability for each of the factors: superordinate, α = .95, monitoring, α = .92, control/debugging, α = .86 and evaluation, α = .87. For factor loadings and item descriptive statistics, see Table 3. On average, students reported a moderate use of monitoring (*M* = 4.51), control/debugging (*M* = 4.51), and evaluation (*M* = 4.70).

#### 2.3.6. Use of JOKS

We also analyzed the JOKs (α = .86) using different calculations. As mentioned in the introduction, we calculated the mean absolute accuracy, gamma, and discrimination (see Schraw 2009 for the formulas). Gamma could not be computed for 9 participants (25% of the sample) since they responded with the same confidence rating for all seven items. Therefore, we did not examine gamma in our analyses. Absolute accuracy ranged from .06 to .57, with a lower score indicating better precision in their judgments, whereas discrimination in this study ranged from −3.75 to 4.50, with more positive scores indicating that students were able to indicate when they knew something.

### 2.4. Procedure

The study took approximately 120 min to complete (see Figure 4 an overview). At the beginning of the study, students were informed that they were going to be videotaped during the experiment and consented to participating in the study. Then, they moved on to complete the pre-test (15 min), followed by the experimenter instructing students to say their thoughts aloud. Then, the experimenter gave the students a sheet of paper with three multiplication problems on it. If students struggled to think aloud while solving problems (i.e., they did not say anything), then the experimenter modeled how to think aloud. Once students completed all three problems and the experimenter was satisfied that they understood how to think aloud (3 min), the experimenter moved onto the learning activity. Students had 15 min to complete the variability learning activity. After the variability activity, students watched a consolidation video (15 min) and worked through a standard deviation activity (15 min). Then, they were asked to complete the task-based questionnaire (10 min). Once the questionnaire was completed, the students had 35 min to complete the post-test. Upon completion of the post-test, students completed several questionnaires, a demographic survey, and then students were debriefed (12 min).

## 3. Results

The first set of analyses examined whether the three measures were related to one another. The second set of analyses evaluated the degree to which the different measures related to learning, transfer, and PFL, providing external reliability for the measurements. Descriptive statistics for each measure are represented in Table 4. For all analyses, alpha was set to .05 and results were interpreted as trending if *p* < .10.

### 3.1. Relation within and across Metacognitive Measures

To evaluate whether the measures revealed similar associations between the different skills both within and across the measures, we used Pearson correlation analyses. See Table 5 for all correlations. Within the measures, we found that there were no associations among the skills in the verbal protocol codes, but there were positive associations between all the skills in the task-based questionnaire (monitoring, control/debugging, and evaluation). For the JOKs, there was a negative association between mean absolute accuracy and discrimination, meaning that the more accurate participants were at judging their confidence (a score closer to zero for absolute accuracy), the more likely they were aware of their correct performance (positive discrimination score). There was also a positive association between the average ratings of the JOKs and discrimination, meaning those who were assigning higher values in their confidence were also more aware of their correct performance.

Across the measures, an interesting pattern emerged. The proportion of monitoring statements was negatively associated with the monitoring questionnaire and the average JOK ratings. However, there was no relationship between the monitoring questionnaire and the average JOK ratings. For the other skills, control/debugging and evaluation questionnaire responses positively correlated with the proportion of evaluation statements. There were also two trends for the monitoring questionnaire, such that it was positively related to the proportion of evaluation statements and the average JOK ratings. Otherwise, there were no other associations.

### 3.2. Relation between Metacognitive Measures and Learning

#### 3.2.1. Learning and Test Performance

The learning materials included the first and second learning activities, and a post-test that included transfer items and a PFL item. For the first learning activity, the scores ranged from 0 to 3 (out of 4) with an average score of 1.6 points (*SD* = .72, 40%). For the second learning activity, the scores ranged between 0 and 2 (out of 5) with an average score of 1.56 points (*SD* = .59; 31%). Given the low performance when solving the second activity and the observation that most students were applying mean deviation to the second activity, instead of inventing a new procedure, we did not analyze these results. For the post-test transfer items, the scores ranged from 1 to 5.67 (out of 6) with an average score of 3.86 points (*SD* = 1.26). We did not include the PFL in the transfer score, as we were particularly interested in examining the relation between the metacognitive measures and PFL. The PFL scores ranged from 0 to 1 (out of 1) with an average score of 0.49 (*SD* = 0.51). For ease of interpretation, we converted student scores for all learning measures into the correct proportion in Table 6.

To evaluate the relation between each metacognitive measure and the learning materials, we used a series of regressions. We used multiple linear regressions to test the amount of variance explained in the first learning activity and post-test performance by each measure. Then, to test the amount of variance explained by each metacognitive measure in the PFL performance, we used multiple logistic regression. In addition to these models, we also regressed the learning outcomes on the most predictive variables from each of the measures and entered them into a competing model to evaluate whether and how much they uniquely contribute to the overall variance.

#### 3.2.2. Verbal Protocols and Learning Outcomes

For verbal protocols, we entered each of the codes into the model. The model predicting performance on the first learning activity explained 14.2% of the variance as indexed by the adjusted *R*^2^ statistic, *F*(3, 40) = 2.21, *p* = .10. Within the model, there was only an effect of monitoring, *β* = −0.37, *t* = −2.51, *p* = .02, VIF = 1.00 (Table 7). The models predicting transfer, *F*(3, 40) = 0.19, *p* = .90, and PFL scores, χ^2^(3, N = 44) = 5.05, *p* = .17, were not significant.

#### 3.2.3. Task-Based Questionnaire and Learning Outcomes

For the task-based questionnaire, we computed two types of models: one with all three metacognitive skills and the other with each metacognitive skill entered separately. Entering all three skills simultaneously led to no significant relations for the first learning activity, *F*(3, 41) = 1.46, *p* = .24, transfer, *F*(3, 41) = 0.15, *p* = .93, or PFL χ^2^(1, N = 45) = 2.97, *p* = .40. However, because the three factors were highly correlated, we entered each factor into three separate models (Kraha et al. 2012).

Entering the skills into separate models revealed a marginal effect of self-reported monitoring, *β* = 0.27, *t* = 1.87, *p* = .07, VIF = 1.00, and self-reported evaluation, *β* = 0.29, *t* = 2.0, *p* = .05, VIF = 1.00, on the first learning activity. The model predicting performance on the first learning activity with self-reported monitoring explained 7.5% of the variance as indexed by the adjusted *R*^2^ statistic, *F*(1, 43) = 3.50, *p* = .07, whereas the model predicting performance on the first learning activity with self-reported evaluation explained 8.5% of the variance as indexed by the adjusted *R*^2^ statistic, *F*(1, 43) = 4.01, *p* = .05. Otherwise, there were no significant relations. Self-reported monitoring and evaluation were not related to performance on transfer, *F*(1, 43) = 0.1, *p* = .75 and *F*(1, 43) = 0.02, *p* = .88), respectively, or PFL scores, χ^2^(1, N = 45) = 0.01, *p* = .91, χ^2^(1, N = 45) = 1.29, *p* = .26), respectively, and self-reported control/debugging had no relation to any of the learning outcomes (learning activity: *F*(1, 43) = 1.52, *p* = .22; transfer: *F*(1, 43) = 0.07, *p* = .79; PFL: χ^2^(1, N = 45) = .69, *p* = .41).

#### 3.2.4. JOKs and Learning Outcomes

The JOK calculations were entered into three separate models for each learning outcome, since they were highly correlated with each other.

*Average ratings*. The model predicting first activity explained 10.4% of the variance as indexed by the adjusted *R*^2^ statistic, *F*(1, 43) = 6.11, *p* = .02, in which there was an effect of average JOK ratings, *β* = 0.35, *t* = 2.47, *p* = .02, VIF = 1.00. The model predicting transfer explained 14.1% of the variance as indexed by the adjusted *R*^2^ statistic, *F*(1, 43) = 7.07, *p* = .01, in which there was an effect of average JOK ratings, *β* = 0.38, *t* = 2.66, *p* = .01, VIF = 1.00. The logistic model predicting PFL scores explained 15.6% of the variance as indexed by the adjusted Nagelkerke *R*^2^ statistic, χ^2^(1, N = 43) = 5.6, *p* < .05. There was an effect of average JOK ratings, B = 4.17, Exp (B) = 64.71, Wald’s χ^2^(1, N = 44) = 4.21, *p* = .04. Thus, higher average JOK ratings were associated with an increase in the likelihood of solving the PFL problem.

*Mean absolute accuracy*. The model predicting first activity explained 4.2% of the variance as indexed by the adjusted *R*^2^ statistic, *F*(1, 42) = 1.85, *p* =.18. The model predicting transfer explained 50.8% of the variance as indexed by the adjusted *R*^2^ statistic, *F*(1, 42) = 43.42, *p* < .001, in which there was an effect of mean absolute accuracy, *β* = −0.71, *t* = −6.59, *p* < .001, VIF = 1.00. The logistic model predicting PFL scores explained 8.9% of the variance as indexed by the adjusted Nagelkerke *R*^2^ statistic, χ^2^(1, N = 43) = 3.03, *p* = .08, in which there was a marginal effect of mean absolute accuracy, B = −4.26, Exp (B) = 0.01, Wald’s χ^2^(1, N = 44) = 2.74, *p* = .098. Thus, increasing mean absolute accuracy (i.e., worse accuracy) was associated with a reduction in the likelihood of solving the PFL problem.

*Discrimination*. The model predicting performance on the first activity explained 0.1% of the variance as indexed by the adjusted *R*^2^ statistic, *F*(1, 42) = 0.05, *p* = .83. The model predicting transfer explained 88.1% of the variance as indexed by the adjusted *R*^2^ statistic, *F*(1, 42) = 318.61, *p* < .001, in which there was an effect of discrimination, *β* = 0.94, *t* = 17.85, *p* < .001, VIF = 1.00. The logistic model predicting PFL scores explained 33.6% of the variance as indexed by the adjusted Nagelkerke *R*^2^ statistic, χ^2^(1, N = 43) = 12.80, *p* < .001, in which there was an effect of discrimination, B = 0.60, Exp (B) = 1.82, Wald’s χ^2^(1, N = 44) = 8.88, *p* = .003. Thus, increasing discrimination was associated with an increased likelihood of solving the PFL problem.

#### 3.2.5. Competing Models

We evaluated the competing models for the learning activity to determine whether constructs from different measurements were predictive of differential variances within these learning outcomes. The models predicting transfer and PFL were not computed, as only the JOKs were predictive. For the model predicting the first learning activity, we regressed it on self-reported evaluation, monitoring statements, and JOK average. The model explained 24.7% of the variance as indexed by the adjusted *R*^2^ statistic, *F*(3, 40) = 4.37, *p* = .009. Within the model, there was a marginal effect of self-reported evaluation, *β* = 0.24, *t* = 1.71, *p* = .095, VIF = 1.03. Otherwise, there were no other significant effects (Table 8).

## 4. Discussion

From these results, we raise some important questions about the measures of metacognitive regulation, specifically those that assess the skills of monitoring, control/debugging, and evaluation. Not only do we find that the task-based questionnaire, verbal protocols, and JOK measures assessing these skills show little relation to one another, but they also predict different learning outcomes. Although these results suggest that these measures are capturing different processes, one aspect of these results suggests that they capture some overlapping variance, such that the different types of measures did not result in a significant model in the competing model for the learning activity. Below, we discuss these results further by first focusing on relation among the measures and their relation to learning outcomes and then turning to their implications and areas for future research.

### 4.1. Relation of Measures

A central goal of this study was to examine the degree to which these different measures of metacognitive regulation relate to each other for a subset of metacognitive skills (monitoring, control/debugging, and evaluation). The results demonstrated that there is little association between the task-based metacognitive regulation questionnaire and the corresponding verbal protocols, suggesting that these measurements are inaccurate, measure different processes than intended, or some combination of the two. For example, self-reported monitoring was negatively related to the monitoring statements. This finding suggests that the more students monitored their understanding, the less likely they were to report doing so on a questionnaire, reflecting a disconnect between what students do versus what they think they do. This misalignment might be particularly true for students who are struggling with the content and are making more monitoring statements. It also implies that students are unaware of the amount they are struggling—or worse, they are aware of it, but when asked about it, they are biased to say the opposite, perhaps because they do not want to appear incompetent. This speculation is also related to the observational finding that when students monitored their understanding, they were more likely to share negative monitoring statements such as “I do not understand this.” Therefore, perhaps a more in-depth analysis of the monitoring statements might provide clarity on the relation between these two measures. Another possibility is a mismatch of monitoring valence across the two measures because the monitoring questionnaire items are almost all positively framed (e.g., “*During the activity*, *I felt that I was gradually gaining insight into the concepts and procedures of the problems*”), whereas the verbal protocols could capture either positive or negative framings. If what is being expressed in the verbal protocols is just monitoring what one does not understand, then we would expect to see a negative correlation such as the one we found. That is, self-reported monitoring is likely to be negatively aligned with negative monitoring statements but potentially not positive monitoring statements. A similar pattern might also be true of the JOK average ratings and the monitoring statements, as they were also negatively associated with each other, especially since the JOKs capture one’s confidence.

The frequency of evaluation statements was associated with self-reported evaluation as well as self-reported control/debugging, which suggests that the different self-reported constructs capture a similar aspect of metacognitive behavior. There was also a trend in which self-reported monitoring was also positively related to evaluation statements. This partial alignment between the questionnaire and verbal protocols might be due to students’ awareness in the moment in which some processes are more explicit (e.g., evaluation) than others (e.g., control/debugging). The lack of differentiation on the questionnaire could also be attributed to students not being very accurate at knowing what they did and did not do during a learning task. This interpretation is consistent with work by Veenman et al. (2003), in which students’ self-reports had little relation to their actual behaviors. Instead, students might be self-reporting the gist of their actions and not their specific behaviors which are captured in the verbal protocols. It is also possible that there could have been more overlap between the two measures if we coded the verbal protocols for the entire set of learning activities that the students were self-reporting about (not just the first learning activity). It is also unclear as to what students were referencing when answering the self-reports. They could have been referencing their behaviors on the most recent task (i.e., the standard deviation activity) in which we did not code for their metacognitive verbalizations.

There was also a trend in which the average JOK ratings were positively related to self-reported monitoring, suggesting that the average JOK ratings reflected some aspects of monitoring that were captured in the questionnaire. Otherwise, there were no associations between the JOKs and the monitoring and evaluation statements or questions. As mentioned earlier, JOKs capture the accuracy of one’s monitoring and evaluating, not just the act of performing the skill or recounting how many times they engaged in an instance. This result reveals that perhaps being able to identify when one engages in the skills is different from gauging whether one is understanding information or self-reporting on whether one was engaged in checking one’s understanding. Another interpretation is that the JOK accuracy might benefit from the additional learning experiences that took place after the verbal protocols (i.e., the consolidation video) and after the questionnaire (i.e., the embedded resource). These additional resources may provide a more comprehensive picture of the learner’s understanding and might have allowed them to resolve some of their misunderstandings. Prior research also shows that students can learn from a test (Pan and Rickard 2018), providing them with additional information to inform their judgments.

The learning activity might have also played a role in the relationship across the different measures. As mentioned, the structured inquiry task allows for more opportunities to engage in metacognition. This opportunity might also allow for instances in which the metacognitive skills are difficult to distinguish, as they might co-occur or overlap with each other. Perhaps if the learning activity were designed to elicit a specific metacognitive behavior, different associations would emerge.

### 4.2. Robust Learning

In terms of learning, we see that students’ self-reported use of monitoring and evaluation has a marginal relation to their performance on the first activity, which provides some external validity for those two components. However, there was not a relation between the self-reports and the transfer or PFL performance. It could be that the monitoring and evaluation components of the questionnaire were able to predict performance specific to the task with which they were based on but not the application of the knowledge beyond the task. This finding suggests that these questionnaire measures are limited in the types of learning outcomes they can predict. It is also important to note the differences between this work and past; here, the questionnaire was task specific and involved a problem-solving activity, whereas other work has looked at more domain-general content and related the questionnaires to achievement. Therefore, it is difficult to know whether the task specific framing of the questionnaire limits its predictability, or the change in assessment, or both.

The low internal reliability of the transfer post-test could have also posed difficulties in examining these analyses, as students were responding very differently across the items. The lack of internal reliability might be attributed to the combination of different types of transfer items within the assessment. Future work could employ an assessment with multiple items per concept and per transfer type (e.g., near versus intermediate) to determine the extent to which the reliability of the test items impacted the results.

As predicted, there was an association between monitoring verbal protocols and performance on the first learning activity. The negative association, as well as the observation that the majority of the metacognitive statements reflected a lack of understanding, aligns well with Renkl’s (1997) findings, in which negative monitoring was related to transfer outcomes. Although monitoring was not a positive predictor, we used a verbal protocol rubric that differs from those who have found positive learning outcomes as we coded for the frequency of the metacognitive statements and not other aspects of a metacognitive event, such as the quality or valence; (e.g., Van der Stel and Veenman 2010). For example, the quality of the metacognitive event can be meaningful and add precision to the outcomes they predict (Binbasaran-Tuysuzoglu and Greene 2015). We did not see an association between the verbal protocols with performance on the transfer or PFL problems. One reason for the lack of relationship might be that the verbal protocols occurred during encoding stage with different materials and were not identical to the retrieval- and application-based materials that were used at the post-test. Although there is no prior work evaluating PFL with verbal protocols, other work evaluating transfer suggests that we would have found some relation (e.g., Renkl 1997). It would be productive for research to explore how different verbal protocol rubrics relate to one another and whether the types of verbal protocols elicited from different tasks result in different relations to robust learning.

Students’ average JOK ratings, absolute accuracy (knowing when they knew something), and discrimination (rating correct items with higher confidence than incorrect items) were strong predictors of performance on transfer and PFL. These relations could be due to the time-contingent and content-dependent aspects of JOKs, as they were tied to the test which occurred after the learning, whereas the verbal protocols and questionnaires were tied to the learning materials and occurred during and after the learning materials, respectively. Regardless, these findings suggest that being able to monitor one’s understanding is important for learning outcomes. Given there was a strong negative relation between the average JOK ratings and monitoring questionnaire and no relationship between the questionnaire and discrimination and absolute accuracy, it also supports that these measures capture different aspects of metacognition. JOKs might be assessing one’s accuracy at identifying their understanding (i.e., monitoring accuracy) whereas the average JOKs and the monitoring questionnaire might be assessing one’s awareness of checking one’s understanding. However, when comparing the average JOK ratings to the monitoring questionnaire on performance for the first learning activity, the average JOKs have a stronger relationship, implying that after a learning experience and consolidation lecture, students are more accurate at recognizing their understanding.

Although prior work has argued that JOKs are domain general (Schraw 1996), we do not find discrimination or absolute accuracy to be predictive of the learning activity; however, the average JOK ratings were predictive. Students who had higher average JOKs performed better on the learning activity, but it did not matter how accurate their JOKs were. However, for transfer and PFL measures, their accuracy in their monitoring did matter. This finding suggests that students’ ability to monitor their understanding might transfer across different learning measures, but their accuracy is more dependent on the actual learning measure. This assumption is consistent with prior work in which students’ monitoring accuracy varied as a function of the item difficulty (Pulford and Colman 1997).

When generating competing models across the metacognitive measures, we were only able to examine one in which we predicted performance on the first activity with evaluation questionnaire, monitoring statements, and JOK average. The overall model was not significant. This finding suggests that they captured shared variances in their relation to learning, but that they are distinctly different in that they were not associated with each other.

### 4.3. Theoretical and Educational Implications

One goal of this study was to explore the relation between different skills and at what level of specificity to describe the constructs. We were able to establish a second-order factor with a task-based survey in which the different skills represented the higher-order factor of metacognitive regulation, but also the unique factors for each skill, such that they were distinguishable. We were also able to distinguish between the different metacognitive skills in the verbal protocols with adequate inter-rater reliability between the two coders and the differential relations the codes had with each other and the learning and robust learning outcomes. The lack of correlation between the verbal protocol codes shows that they are not related to each other and suggests that they are capturing different skills. This finding is further supported when predicting learning outcomes, as the verbal protocol codes are related to different types of learning outcomes. This work highlights the need for future theory building to incorporate specific types of metacognitive skills and measures into a more cohesive metacognitive framework. Doing so would inform both future research examining how these processes operate, as well as educators who want to understand whether there are particular aspects of metacognition that their students could use more or less support in using.

This work also has practical implications for education. Although verbal protocols provide insight into what participants were thinking, they were least predictive of subsequent learning performance. However, the utility in using verbal protocols in classroom settings is still meaningful and relevant in certain situations. Of course, a teacher could not conduct verbal protocols for all their students, but it could be applied if they were concerned about how a particular student was engaging in the problem-solving process. In this case, a productive exercise might be to ask the student to verbalize their thoughts as they solve the problem and for the teacher to take notes on whether there are certain metacognitive prompts that may help guide the student during their problem-solving process.

The task-based questionnaire and the metacognitive judgment measures, which are more easily applied to several students at one time and thus are more easily applied in educational contexts, had stronger relations to learning outcomes. Given that the JOKs in this study were positively related to multiple learning outcomes, it might have more utility in the classroom settings. The use of these JOKs will allow teachers to measure how well students are able to monitor their learning performance. To compliment this approach, if teachers want to understand whether their students are engaging in different types of metacognitive skills as they learn the content in their courses, then the use of the task-based questionnaire could readily capture which types of metacognitive skills they are employing. The use of these measures can be used in a way that is complimentary, given the goals of the teacher.

### 4.4. Future Research

This work examines a subset of metacognitive measures, but there are many more in the literature that should be compared to evaluate how metacognitive regulation functions. Given the nature of the monitoring examined in this work, it would be particularly interesting to examine how different metacognitive judgments such as judgments of learning relate to the monitoring assessed by the verbal protocols and the questionnaire. Kelemen et al. (2000) provide evidence that different metacognitive judgments assess different processes, so we might expect to find different associations. For example, perhaps judgments of learning are more related to monitoring statements than JOKs. Judgments of learning have a closer temporal proximity to the monitoring statements and target the same material as the verbal protocols. In contrast, JOKs typically occur at a delay and assess post-test materials that are not identical to the material presented in the learning activity. In this work, we were not able to capture both judgments of learning and JOKs because the learning activity did not allow for multiple measures of judgments of learning. Therefore, if a learning activity allowed for more flexibility in capturing multiple judgments of learning, then we might see different relations emerge due to the timing of the measures.

Future work could also explore the predictability the task-based questionnaire has over other validated self-report measures such as a domain-based adoption of the MAI or MSLQ. It would also be interesting to examine how these different measures relate to other external factors as predicted by theories of self-regulated learning. Some of these factors include examining the degree to which the task-based questionnaire, JOKs, and verbal protocols relate to motivational aspects such as achievement goal orientations, as well as more cognitive sense-making processes such as analogical comparison and self-explanation. Perhaps this type of research would provide more support for some self-regulated learning theories over others given their hypothesized relationships. More pertinent to this line of work, this approach has the potential to help refine theories of metacognitive regulation and their associated measures by providing greater insight into the different processes captured by each measure and skill.

## Figures and Tables

**Table 1:** Overview of the metacognitive regulation measurements.

Measurement	Metacognitive Skill	Timing	Framing of the Assessment	Analytical Measures	Predicted Learning Outcome
Verbal Protocols	Monitoring, Control/Debugging, and Evaluating	Concurrent	Task based	Inter-rater reliability, Cronbach’s alpha	Learning, transfer, and PFL
Questionnaires	Monitoring, Control/Debugging, and Evaluating	Retrospective	Task based	Second-Order CFA, Cronbach’s alpha	Learning, transfer, and PFL
Metacognitive Judgments—JOKs	Monitoring and Monitoring Accuracy	Retrospective	Test items	Cronbach’s alpha, Average, Mean Absolute accuracy, Gamma, and Discrimination measures	Learning, transfer, and PFL

**Table 2:** Verbal coding rubric.

Code Type	Definition	Transcript Examples
Monitoring	Checking one’s understanding about what the task is asking them to do; making sure they understand what they are learning/doing.	“I’m gonna figure out a pretty much the range of them from vertically and horizontally? I’m not sure if these numbers work (inaudible)”. “That doesn’t make sense”.
Control/Debugging	An action to correct one’s understanding or to enhance one’s understanding/progress. Often involves using a different strategy or rereading.	“I’m re-reading the instructions a little bit” “So try a different thing”.
Conceptual Error Correction	A statement that reflects an understanding that something is incorrect with their strategy or reflects noticing a misconception about the problem.	“I’m thinking of finding a better system because, most of these it works but not for Smythe’s finest because it’s accurate, it’s just drifting”.
Calculation Error Correction	Noticing of a small error that is not explicitly conceptual. Small calculator errors would fall into this category.	“4, whoops”.
Evaluation	Reflects on their work to make sure they solved the problem accurately. Reviews for understanding of concepts as well as reflects on accurate problem-solving procedures such as strategies.	“Gotta make sure I added all that stuff together correctly”. “Let’s see, that looks pretty good”. “Let’s check the match on these.”

**Table 3:** Descriptive statistics and factor loading for questionnaire items.

Item	Original Construct	[Min, Max]	*M (SD)*	Standardized Factor	Residual Estimate	Variance
***Monitoring***				***.90***	***.94***	
During the activity, I found myself pausing to regularly to check my comprehension.	MAI (Schraw and Dennison 1994)	[1, 7]	4.20 (1.78)	.90	.81	0.19
During the activity, I kept track of how much I understood the material, not just if I was getting the right answers.	MSLQ Adaptation (Wolters 2004)	[1, 7]	4.18 (1.60)	.83	.69	0.31
During the activity, I checked whether my understanding was sufficient to solve new problems.	Based on verbal protocols	[1, 7]	4.47 (1.59)	.77	.59	0.41
During the activity, I tried to determine which concepts I didn’t understand well.	MSLQ (Pintrich et al. 1991)	[1, 7]	4.44 (1.65)	.85	.73	0.27
During the activity, I felt that I was gradually gaining insight into the concepts and procedures of the problems.	AILI (Meijer et al. 2013)	[2, 7]	5.31 (1.28)	.75	.56	0.44
During the activity, I made sure I understood how to correctly solve the problems.	Based on verbal protocols	[1, 7]	4.71 (1.46)	.90	.80	0.20
During the activity, I tried to understand why the procedure I was using worked.	Strategies (Belenky and Nokes-Malach 2012)	[1, 7]	4.40 (1.74)	.78	.62	0.39
During the activity, I was concerned with how well I understood the procedure I was using.	Strategies (Belenky and Nokes-Malach 2012)	[1, 7]	4.38 (1.81)	.74	.55	0.45
***Control/Debugging***				***.81***	***.66***	
During the activity, I reevaluated my assumptions when I got confused.	MAI (Schraw and Dennison 1994)	[2, 7]	5.09 (1.58)	.94	.89	0.11
During the activity, I stopped and went back over new information that was not clear.	MAI (Schraw and Dennison 1994)	[1, 7]	5.09 (1.54)	.65	.42	0.58
During the activity, I changed strategies when I failed to understand the problem.	MAI (Schraw and Dennison 1994)	[1, 7]	4.11 (1.67)	.77	.60	0.40
During the activity, I kept track of my progress and, if necessary, I changed my techniques or strategies.	SMI (O’Neil and Abedi 1996)	[1, 7]	4.51 (1.52)	.89	.79	0.21
During the activity, I corrected my errors when I realized I was solving problems incorrectly.	SMI (O’Neil and Abedi 1996)	[2, 7]	5.36 (1.35)	.50	.25	0.75
During the activity, I went back and tried to figure something out when I became confused about something.	MSLQ (Pintrich et al. 1991)	[2, 7]	5.20 (1.58)	.87	.75	0.25
During the activity, I changed the way I was studying in order to make sure I understood the material.	MSLQ (Pintrich et al. 1991)	[1, 7]	3.82 (1.48)	.70	.49	0.52
During the activity, I asked myself questions to make sure I understood the material.	MSLQ (Pintrich et al. 1991)	[1, 7]	3.60 (1.59)	.49	.25	0.76
REVERSE During the activity, I did not think about how well I was understanding the material, instead I was trying to solve the problems as quickly as possible.	Based on verbal protocols	[1, 7]	3.82 (1.72)	.54	.30	0.71
***Evaluation***				***.84***	***.71***	
During the activity, I found myself analyzing the usefulness of strategies I was using.	MAI (Schraw and Dennison 1994)	[1, 7]	5.02 (1.55)	.48	.23	0.77
During the activity, I reviewed what I had learned.	Based on verbal protocols	[2, 7]	5.04 (1.40)	.57	.33	0.67
During the activity, I checked my work all the way through each problem.	IMSR (Howard et al. 2000)	[1, 7]	4.62 (1.72)	.94	.88	0.12
During the activity, I checked to see if my calculations were correct.	IMSR (Howard et al. 2000)	[1, 7]	4.73 (1.97)	.95	.91	0.09
During the activity, I double-checked my work to make sure I did it right.	IMSR (Howard et al. 2000)	[1, 7]	4.38 (1.87)	.89	.79	0.21
During the activity, I reviewed the material to make sure I understood the information.	MAI (Schraw and Dennison 1994)	[1, 7]	4.49 (1.71)	.69	.48	0.52
During the activity, I checked to make sure I understood how to correctly solve each problem.	Based on verbal protocols	[1, 7]	4.64 (1.57)	.86	.75	0.26

Note. The bolded italics represents each of the three factors with their respective items listed below each factor.

**Table 4:** Descriptive statistics for each measure.

Measure	Variable	*N*	Min	Max	*M*	*SE*	*SD*
Verbal Protocols	Monitoring	44	0.00	0.29	0.05	0.01	0.06
	Control/Debugging	44	0.00	0.06	0.01	0.002	0.02
	Evaluation	44	0.00	0.16	0.04	0.01	0.04
Questionnaire	Monitoring	45	1.13	6.75	4.51	0.19	1.29
	Control/Debugging	45	2.33	6.44	4.51	0.16	1.08
	Evaluation	45	2.14	7.00	4.70	0.19	1.28
JOKs	Mean	45	2.00	5.00	4.31	0.09	0.60
	Mean Absolute Accuracy	45	0.06	0.57	0.22	0.02	0.13
	Discrimination	45	−3.75	4.5	1.43	0.33	2.21

Note. To control for the variation in the length of the verbal protocols across participants, the verbal protocol measures were calculated by taking the total number of times the specified verbal protocol measure occurred by a participant and dividing that by the total number of utterances that participant made during the learning activity.

**Table 5:** Correlations between the task-based questionnaire, verbal protocols, and judgments of knowing.

Variable		1	2	3	4	5	6	7	8	9
VPs	1. Monitoring	-	.09	.01	−.36 *	−.10	−.16	−.41 *	− .07	−.14
	2. Control/Debugging		-	.16	.12	−.08	.14	−.16	.03	−.08
	3. Evaluation			-	.29 ^†^	.31 *	.37 *	−.10	.02	.01
Qs	4. Monitoring				-	.73 **	.73 **	.26 ^†^	.06	.02
	5. Control/Debugging					-	.65 **	.02	−.02	−.03
	6. Evaluation						-	.15	.11	−.09
JOKs	7. Average							-	.14	.39 **
	8. Mean Absolute Accuracy								-	− .76 **
	9. Discrimination									-

Note. VPs = Verbal Protocols, Qs = Questionnaire, JOKs = Judgments of Knowing, ^†^ = *p* < .10, * = *p* < .05, and ** *p* < .01.

**Table 6:** Descriptive statistics for each learning measure.

Measure	*N*	Min	Max	*M*	*SE*	*SD*
First Learning Activity	45	0.00	0.75	0.40	0.03	0.18
Transfer	45	0.17	0.94	0.64	0.03	0.21
PFL	45	0.00	1.00	0.49	0.08	0.51

**Table 7:** Multiple linear regression model predicting performance on the first activity with verbal protocols.

Variable	*β*	*t*	*p*	VIF
Monitoring statements	−0.37	−2.51	.02 *	1.01
Control/Debugging statements	−0.05	−0.32	.75	1.03
Evaluation statements	−0.03	−0.17	.87	1.02
Constant		10.06	<.001 ***	

Note. * = *p* < .05 and *** *p* < .001.

**Table 8:** Multiple linear regression model predicting performance on the first activity with self-reported evaluation, monitoring statements, and JOK average.

Variable	*β*	*t*	*p*	VIF
Self-reported Evaluation	0.24	1.71	.095	1.03
Monitoring Statements	−0.24	−1.60	.12	1.22
JOK Average	0.23	1.53	.13	1.21
Constant		−0.08	.93	

## Data Availability

Summary levels of the data presented in this study are available on request from the corresponding author. The data are not publicly available to protect the privacy of the participants.
